# Memory for abstract control states does not decay with increasing retrieval delays

**DOI:** 10.1007/s00426-023-01870-4

**Published:** 2023-08-24

**Authors:** Moritz Schiltenwolf, Andrea Kiesel, Christian Frings, David Dignath

**Affiliations:** 1 Department of Psychology, University of Tübingen, Schleichstrasse 4, 72076 Tübingen, Germany; 2 University of Freiburg, Freiburg, Germany; 3 University of Trier, Trier, Germany

## Abstract

Recent studies have suggested that abstract control states (i.e., internal attentional states independent from concrete stimuli and responses) can be stored in episodic memory and retrieved subsequently. However, the duration of such a control state memory remains unclear. Previous research has found a quick and complete decay for stimulus–response bindings after 2000–5000 ms. Here, we tested a possible decay of control state bindings with retrieval delays of 2000, 3000, or 5000 ms. Five preregistered experiments used a confound-minimized prime-target task to measure the congruency sequence effect (CSE) separately for trials in which a nominally irrelevant context feature changed or repeated across trials. Analyses of the individual experiments did not result in conclusive evidence. A mega-analysis integrating the data of all experiments (*N*_*total*_ = 326) replicated evidence for binding and retrieval of control states, in that larger CSEs were found for context repetition trials. Importantly, Bayesian analysis indicated that this effect was not modulated by the length of retrieval delay. While this finding suggests that bindings of abstract control states can be relatively robust, we also discuss possible limitations of the present research.

**Supplementary Information:**

The online version contains supplementary material available at 10.1007/s00426-023-01870-4.

## Introduction

Human behavior is highly context specific. Seeing the orange lights of roadworks does not bother us as pedestrians, but it immediately calls for more attention when we are driving a car. Theoretical approaches to human action control have acknowledged this by emphasizing the role of memory in adaptive action control (Frings et al., 2020; Henson et al., 2014; Hommel et al., 2001). More specifically, it is assumed that perceived (contextual) stimuli and executed responses are stored in episodic memory in so-called event-files that bind together co-occurring features for a short duration (Hommel, 2004). Repetition of previously encountered features will retrieve other co-occurring features from memory (Colzato et al., 2006, e.g.; Hommel et al., 2014; see also Schumacher & Hazeltine, 2016). This approach has been successful in explaining a wide range of effects, such as action-effect anticipation (Kunde, 2001; Kunde et al., 2002), stimulus–response translation (Frings et al., 2007; Hommel, 1998), negative priming (Frings et al., 2015; Rothermund et al., 2005) and task switching (Kiesel et al., 2010; Koch et al., 2018).

Because situations are often complex and require control over an ever-changing series of stimuli and possible responses, the question arises as to whether binding and retrieval is limited to concrete stimulus–response links or whether it can also account for behavior that relies on abstract representations (see also Singh et al., 2019). A canonical case of abstraction is cognitive control, which refers to a set of superordinate functions that allow the maintenance of current goals and task sets independent of specific stimuli or responses (e.g. Botvinick & Braver, 2015). Cognitive control functions have often been assessed with response-interference tasks. These tasks manipulate the match between task-relevant target and task-irrelevant distractor dimensions. For incongruent trials, in which the target and distractor indicate different responses, performance is impaired (longer RTs and more errors) compared to congruent trials, in which the target and distractor indicate the same response and thereby facilitate performance.

Interestingly, it has been suggested that the relative weighting of target and distractor information can be flexibly adapted according to recent experiences (see e.g. Egner, 2017). For instance, previous incongruent stimuli decrease the influence of current distractors, whereas previous congruent stimuli increase the impact of current distractors. This effect, known as the congruency sequence effect (CSE), has been attributed to dynamic changes in attention (Botvinick et al., 2001). According to this account, conflict in the previous trial serves as a learning signal that strengthens relevant and suppresses irrelevant processing pathways, which reduces the relative impact of conflicting information in the current trial (but see Lamers & Roelofs, 2011 for evidence that control is driven by congruent trials). However, in this conflict monitoring account, it remained unclear how the information about recent conflict experiences, i.e., the learning signal, is maintained in the time interval between trials. To fill this gap, a *short-term memory for experienced conflict* was proposed as a maintenance system for the learning signal (Mansouri et al., 2007, 2009). This idea has been revisited by more recent binding accounts suggesting that memory stores a snapshot of the attentional state after control exertion (Abrahamse et al., 2016; Crump, 2016; Egner, 2014; Schumacher & Hazeltine, 2016). For instance, the *Binding and Retrieval in Action Control* (BRAC) framework proposes that, similar to bindings of concrete features such as stimuli and responses, ‘instances’ of abstract control parameters (e.g., attentional weights of stimulus and response codes) are integrated into an event-file and can be retrieved under appropriate conditions (Frings et al., 2020). We refer to such internal states as abstract because they modulate the activation of stimuli and responses independently from the concrete perceptual and response features.

This mnemonic control hypothesis has received support from neurophysiological and behavioral studies. For instance, Jiang et al., (2015; see also Jiang et al., 2020) showed that the CSE could be attributed to increased activity in the anterior hippocampus, a region that has been strongly associated with the integration and subsequent retrieval of bindings via pattern completion (Horner et al., 2015; Rolls, 2013). More direct evidence for memory-based control comes from behavioral studies that manipulated retrieval conditions, for instance, by changing the availability of retrieval cues. More specifically, because abstract control states co-occur with the perception of stimuli or the execution of actions in the previous trial, repetition of stimuli or responses in the next trial act as retrieval cues recollecting related control states from memory. Evidence comes from studies that presented a nominally irrelevant context feature that could either repeat or change across trials and reported increased CSEs for context-repetition compared to context-change trials, possibly because context-repetition facilitated retrieval of control states (e.g. Atalay & Inan, 2017; Braem et al., 2014; Kreutzfeldt et al., 2016; Scherbaum et al., 2011; Spapé & Hommel, 2008).

However, in these studies, the lack of experimental control over transitions between specific stimuli and responses posed a challenge that made it difficult to differentiate the effects of control bindings from possible effects of stimulus–response bindings (Hommel et al., 2004). To address this issue, Dignath et al., (2019; see also Grant et al., 2021) implemented a ‘confound-minimized’ design with different stimulus and response sets for even and odd trial numbers. This design ensured that stimuli and responses did not repeat across trials. At the same time, a nominally irrelevant context feature (e.g., whether a number was presented as a digit or a word) could change or repeat across trials. Importantly, unlike paradigms in which contingencies between context and congruency levels are learned, context did not provide information about task demands (Crump et al., 2006). They assumed that on each trial the adopted control state and the displayed context feature would be bound into an event-file (e.g., in an incongruent trial in which the stimuli were displayed as number word, a control state weighting target over distractor information and the number word format become bound in an event-file). Repetition of the context across two trials should result in a retrieval of the previously bound control state. CSEs, serving as markers for the strength of previous control adaptations on current behavior, were larger on context repetition trials than on context change trials. Importantly, these findings could not be attributed to stimulus–response memory, as stimulus and response repetitions were avoided across trials (see Jiménez & Méndez, 2013; Weissman et al., 2014). Additional evidence supporting the effects of control bindings comes from similar studies applying confound-minimized designs to response interference tasks with other contexts such as modality (Grant et al., 2020), task structure (Dignath et al., 2021) or location (Dignath & Kiesel, 2021).

### The present research

The present study examined the temporal stability of bound control states. Previous research on binding and retrieval of stimulus–response bindings suggested that event-files decay rather quickly. For instance, Hommel and Frings (2020) found that the aftereffects of stimuli and response codes gradually decreased with longer intertrial intervals (ITIs). This suggests that temporal delays impair retrieval, possibly because event-files that link stimulus–response codes disintegrate over time (Frings, 2011; Frings et al., 2020; Hommel & Colzato, 2004; for response-outcome bindings see Moeller et al., 2016; for neural evidence see Pastötter et al., 2020). The only documented exceptions to such a rapid disintegration are bindings between sequential actions (Moeller & Frings, 2021) and bindings between actions and action effects (Herwig & Waszak, 2012). Both studies showed that ITIs up to 6 s did not impact the aftereffects of previous trial action codes. To account for their surprising finding, the authors speculated that bindings might serve different functions. Following research on hierarchical action representations (Cooper & Shallice, 2006; see Lashley, 1952), Moeller and Frings (2021) suggested that response–response bindings might enable the formation of complex action representations. For such higher-level representations, temporal stability is relevant because these representations merge temporally distant events. However, at the level of stimulus representations, quick disintegration of stimulus–response bindings seems more advantageous to prevent interference between individual episodes (Hommel & Frings, 2020). For control bindings it remains unclear which time course is to be expected. Hitherto, only action bindings have been shown to be temporally stable (Herwig & Waszak, 2012; Moeller & Frings, 2021). However, since the confound-minimized design eliminates binding of response codes, one might predict that the context-transition effects on the CSE (c-CSE) becomes smaller with increasing delays, e.g., because representations of perceptual context features decay over time (e.g., Hommel & Frings, 2020). Alternatively, one might speculate that similar to action bindings, control bindings might support complex behavior by balancing in how far attentional settings from previous episodes generalize to new episodes (e.g., Badre et al., 2021). Indeed, a previous study demonstrated that in the confound-minimized design CSEs are robust against time delays of up to 9 s (Schiltenwolf et al., 2022). In this study features like format, location, or modality were held constant, and thus each trial provided conditions that should facilitate the retrieval of control states from the previous trial. Consequently, temporally robust CSEs in this study might reflect control state retrieval. Based on this perspective, one would assume that c-CSE in the present research—which allow a more direct assessment of control state retrieval—are also unaffected by time delays.

In this study, we aim to examine the temporal durability of abstract control state bindings are. We conducted a series of five preregistered, highly similar experiments in which binding and retrieval of abstract control states could be inferred using a confound-minimized prime-target task. This design eliminates the influences of stimulus–response bindings across sequentially presented trials. Furthermore, we introduced a nominally task-irrelevant context that could either repeat or change across trials. We predicted larger CSEs in context-repetition compared to context-change trials, based on our assumption that control states become bound to the context. Our prediction follows the reasoning that context-repetition trials provide better retrieval conditions than context-change trials, thereby facilitating control state retrieval and leading to stronger control adaptations that are reflected in the size of the CSE. To examine the temporal stability of control bindings, we administered blocks with short and longer ITIs. If control bindings exhibit a time course similar to stimulus–response bindings, we would anticipate smaller c-CSEs in blocks with long ITIs compared to blocks with short ITIs. Conversely, if control bindings are resistant against temporal decay, akin to action bindings, we would expect no difference between c-CSEs in blocks with long and short ITIs. To evaluate these competing predictions, we used Bayesian inference. Across the experiments, we adjusted three task components to maximize the differences between the critical conditions: First, to put the durability of control state bindings to a stronger test, we increased the ITI durations across experiments (Exp. 1: 2000 ms; Exp. 2 and 3: 3000 ms; Exp. 4 and 5: 5000 ms). Second, Experiment 3 employed an unfilled ITI, based on previous research indicating that bindings decay faster during unfilled intervals (Hommel & Frings, 2020). Finally, in Experiment 5, we added additional context features (Exp. 1–4: Stimulus format; Exp. 5: Stimulus format, stimulus color, and response hand). By enhancing the discriminability between the two varying context levels, we tried to foster the c-CSE measurement.

## Experiments 1–5

### Methods

Because all five experiments were highly similar, we will describe them together to avoid redundancies. The hypotheses, procedures, outlier criteria, methods, and planned analyses of each experiment were preregistered on the Open Science Framework (OSF, osf.io/k8752/registrations). Raw data, scripts for the experiments, and analyses are available on OSF.

#### Participants

We collected data from 326 participants (161 female, 152 male, 3 diverse, 10 did not provide gender information; age mean = 29, range: 18–72) in five experiments (N_1_ = 45, N_2_ = 60, N_3_ = 60, N_4_ = 61, N_5_ = 100). All participants were right-handed and German-speaking. Experiment 1 was conducted at the lab of the University of Freiburg testing a student sample. All other experiments were online experiments, and participants were recruited via Prolific (Palan & Schitter, 2018). The sample size for Experiment 1 was based on a power analysis using the tool G*Power (Faul et al., 2007). We opted for a test power of 1 − β = 0.90, an alpha-error probability of α = 0.05 and an effect size of ηp2 = 0.18, which was reported for the c-CSE in the study of Dignath et al. (2019). Sample sizes of Experiments 2–5 all exceeded the calculated sample size of Experiment 1 and were determined using *Sequential Bayes factors* (Schönbrodt et al., 2016).^1^

Participants with excessive error rates (≥ 75%) or error rates higher than 3 SD from that experiment’s sample mean were excluded and replaced (see Table 1).

**Table 1:** Data exclusion at the participant and trial levels

Experiment	1	2	3	4	5
Participant level					
Error rate > 75%	0	0	0	0	1
Error rate deviating > 3 SD from sample mean	0	1	1	1	1
Trial level					
First trial of each block	0.8%	0.8%	0.8%	0.8%	0.4%
Trials following error trials	7.1%	5.6%	5.9%	5.8%	7.8%
Error trials (RT analysis only)	7.2%	5.6%	5.9%	5.7%	7.8%
RT > 3 SD from participant’s sample mean (RT analysis only)	1.3%	1.4%	1.4%	1.4%	1.1%

#### Task and stimuli

**Fig. 1:**
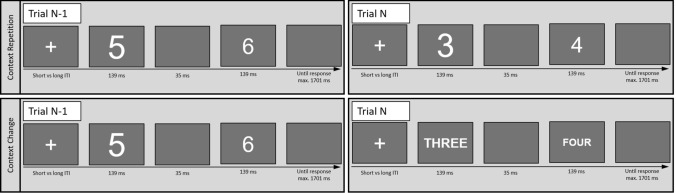
Example trial sequences. *Note*: After presentation of a fixation cross, a distractor (1st stimulus, larger size) and a target (2nd stimulus, smaller size) were presented sequentially. Both distractor and target were presented either as a digit or as a word. This manipulation of stimulus format served as a nominally irrelevant context feature that could either repeat (upper panel) or change (lower panel) across consecutive trials (in Experiment 5, font color and response hand were added as context features). Participants were instructed to respond to the target (2nd stimulus) only. The numbers in the word format were presented in German and are translated into English for this figure.

The experiment was programmed in JavaScript using the library jsPsych (Leeuw, 2015) and closely followed the paradigm of Dignath et al. (2019). Each trial included the presentation of a fixation cross, a distractor stimulus, a blank, a target stimulus, and a response window (see Fig. 1). The distractor was displayed for 139 ms, followed by a blank screen for 35 ms and the target for 130 ms. In Experiments 1–4, distractors and targets were numbers between ‘3’ and ‘6’. In Experiment 5, they were numbers between ‘1’–‘4’ and ‘6’–‘9’. In congruent trials, the target stimulus was identical to the distractor stimulus but different in incongruent trials. In every trial, the target stimulus was presented slightly smaller than the distractor stimulus. After target presentation, a blank response window followed, which was terminated on response or after a maximum of 1701 ms. Participants were instructed to respond to the target stimulus by pressing the corresponding number button on the keyboard. In Experiments 1–4, participants used only their right hand (‘3’: index finger, ‘4’: middle finger, ‘5’: ring finger, ‘6’: little finger). In Experiment 5, participants reacted with their left hand to number stimuli in the range from ‘1’ to ‘4’ (‘1’: little finger, ‘2’: ring finger, ‘3’: middle finger, ‘4’: index finger) and with their right hand to number stimuli in the range from ‘6 to ‘9’ (‘6’: index finger, ‘7’: middle finger, ‘8’: ring finger, ‘9’: little finger). If no or an incorrect response was registered, a red screen was displayed as error feedback for 201 ms. Trials were separated by a delay, i.e., the ITI, which was either ‘short’ or ‘long’. In the short ITI condition, the fixation cross was shown for 250 ms, while it was presented for 2000 ms (Experiment 1), 3000 ms (Experiment 2) or 5000 ms (Experiments 4 and 5) in the long ITI condition. In the long ITI condition of Experiment 3, a blank screen was shown for 2750 ms, followed by a fixation cross shown for 250 ms (resulting in a total ITI of 3000 ms).

Additionally, we introduced a context manipulation. Distractor and target stimuli were displayed in either an Arabic digit format (e.g., ‘3’) or as the corresponding German word in capital letters (e.g., ‘DREI). In Experiment 5, we further expanded the context manipulation by introducing additional features of font color and response hand. For instance, one context level could consist of digits, displayed in orange font color requiring participants to respond with their left hand, while the other context level comprise number words, displayed in blue font color, with participants responding with their right hand. Distractor and target would always be presented in the same context.

#### Procedure

After providing informed consent, task instructions were displayed. The participants were instructed to respond as fast and as accurately as possible and to respond with their right hand only. If the error rate exceeded 40% in the first ten trials of training, instructions were provided again. If participants failed this accuracy test again, the experiment was terminated.

To avoid confounds of stimulus–response memory (e.g., full or partial stimulus and response repetitions, negative priming or contingency learning), we used a confound-minimized design with two different stimulus–response subsets alternating across (see e.g. Jiménez & Méndez, 2013; Schmidt, 2013; Schmidt & Weissman, 2014; Spinelli et al., 2019) trials so that even trials would use different stimulus–response subsets than odd trials. In each block, each of the responses was paired two times with each level of congruency, previous congruency, context, and previous context, resulting in a total of 128 trials per block. After a training block, participants performed eight experimental blocks. The ITI condition in the first block was randomly chosen, alternating from block to block thereafter. The ITI condition in the first block was randomized per participant. Participants were compensated with ca. 5 £/hr.

### Analysis and results

We decided to adjust the preregistered analysis plan by switching from a frequentist to a Bayesian approach (see *Open science and transparency*). Before the test of our main analysis, we successfully validated that the paradigm produced CSEs (see *Appendix f*or the corresponding analyses; see also Table 2).

**Table 2:** CSEs in RTs (ms) and error rates (%) and effects of context-transition on the CSE for all five experiments separated

Experiment	CSE in RTs (ms)					CSE in error rates (%)				
	1	2	3	4	5	1	2	3	4	5
Short ITI duration										
Context repetition	48	52	46	33	27	1.2	1.0	2.0	1.4	1.5
Context change	35	41	32	24	23	3.1	1.0	1.7	1.5	0.9
Context-transition effect (c-CSE)	12	11	13	9	4	− 2.0	0.0	0.3	0.0	0.6
Long ITI duration										
Context repetition	40	34	32	32	31	2.4	1.8	0.3	0.3	0.9
Context change	34	29	37	23	12	3.1	− 1.3	− 0.4	0.3	1.0
Context-transition effect (c-CSE)	6	5	− 5	9	20	− 0.8	3.1	0.7	0.0	− 0.1

CSEs were calculated as (RT_incongruent_ − RT_congruent_)_previous trial incongruent_ − (RT_incongruent_ − RT_congruent_)_previous trial congruent_

To test our main hypothesis, we conducted a Bayesian ANOVA with the within factors of context transition [repetition vs. change] and ITI duration [short vs. long] and participants as random factors with CSE scores as the dependent variables. The CSE score indicates the difference between the congruency effect after previously congruent trials and the congruency effect after previously incongruent trials. It was calculated per participant and condition as CSE = (mean RT_con→inc_ − mean RT_con→con_) − (mean RT_inc→inc_ − mean RT_inc→con_). This analysis was repeated with mean error rates as the dependent variable.

With this analysis approach, we tested the hypothesis that the size of c-CSEs is reduced for longer ITIs. Under H1, we expected reduced c-CSEs for longer ITI conditions relative to shorter ITI conditions. Statistically, H1 predicts a two-way interaction between context transition and ITI duration. Bayes factors were calculated as ${BF}_{10} = \frac{p(data|H1)}{p(data|H0)}$ if *BF*_10_ > 1 and as ${BF}_{01} = \frac{p(data|H0)}{p(data|H1)}$ if *BF*_10_ < 1. Thus, *BF*_10_ indicates the likelihood ratio of the probability that the data would occur under H1 compared to the probability that the data would occur under H0 (e.g., *BF*_10_ = 3 indicates that it is three times as likely to observe the data under the assumption of the H1 model compared to the H0 model), whereas *BF*_01_ indicates the inverse (e.g., *BF*_01_ = 3 indicates that it is three times as likely to observe the data under the assumption of the H0 model compared to the H1 model). In all analyses, Bayes factors for main effects were calculated against an intercept model for H0 (e.g., for the main effect of context transition: H1 model = CSE ~ context transition + participant; H0 model = CSE ~ participant). Bayes factors for interactions were calculated by comparing posterior probabilities for a model including main effects and the interaction term against a model including only main effects but no interaction term (e.g., for the interaction between context transition and ITI duration: H1 model = CSE ~ context transition + ITI duration + context transition: ITI duration + participant; H0 model = CSE ~ context transition + ITI duration + participant). We used the standard prior distribution for fixed effects of.5 for all analyses. *BF*_10_ < 3 and *BF*_01_ < 3 are considered indecisive. Error percentages of the Bayes factor estimated with 10,000 iterations of Monte Carlo sampling are reported (a Bayes factor of 10 with an error percentage of 50% can be expected to fluctuate between 5 and 15).

In accordance with our preregistration, we excluded the first trial of each block and all trials following error trials. For RT analysis, we also removed all error trials and trials with RTs deviating more than 3 SD from this participant’s conditional mean RT (see Table 1).

The results of the analyses of each individual experiment are described in Table 3.

**Table 3:** Resulting Bayes factors resulting from the Bayesian ANOVAs conducted on mean RTs and mean error rates of each experiment

Experiment	1	2	3	4	5
RTs					
Context transition	*BF*_01_ = 1.769 (± 1.27%)	*BF*_*01*_ = *2.793 *(± *1.29%*)	***BF***_***01***_** = *5.559 *(± *2.67%*)**	*BF*_*01*_ = *1.553 *(± *1.32%*)	*BF*_*10*_ = *2.073 *(± *1.28%*)
ITI duration	***BF***_**01**_** = 4.746 (± 1.84%)**	***BF***_**10**_** = 4.727 (± 1.86%)**	***BF***_**01**_** = 5.603 (± 1.04%)**	***BF***_**01**_** = 7.085 (± 1.76%)**	***BF***_**01**_** = 6.714 (± 1.81%)**
Two-way interaction	*BF*_01_ = 1.789 (± 53.24%)	*BF*_01_ = 2.121 (± 52.46%)	*BF*_10_ = 1.421 (± 1.27%)	*BF*_01_ = 2.340 (± 53.18%)	*BF*_10_ = 1.241 (± 53.39%)
Error rates					
Context transition	*BF*_01_ = 2.091 (± 0.83%)	*BF*_10_ = 1.788 (± 0.83%)	***BF***_**01**_** = 5.216 (± 6.65%)**	***BF***_**01**_** = 7.328 (± 0.84%)**	***BF***_**01**_** = 8.703 (± 0.84%)**
ITI duration	***BF***_**01**_** = 4.993 (± 1.04%)**	***BF***_**01**_** = 3.749 (± 1.02%)**	***BF***_**10**_** = 4.021 (± 1.51%)**	*BF*_01_ = 2.549 (± 1.03%)	***BF***_**01**_** = 8.739 (± 1.03%)**
Two-way interaction	***BF***_**01**_** = 3.307 (± 10.33%)**	*BF*_10_ = 2.778 (± 10.35%)	***BF***_**01**_** = 5.000 (± 6.98%)**	***BF***_**01**_** = 4.672 (± 10.52%)**	***BF***_**01**_** = 5.100 (± 10.63%)**

Subscript indicates whether it is evidence in favor of the H1 (BF_10_) or the H0 (BF_01_). Decisive evidence is printed in bold. In brackets, the Bayes factor error percentage is provided

## Discussion experiments 1–5

Experiments 1–5 tested whether the c-CSE decreases with increased ITIs. Across the experiments, we varied the duration of the longer ITI (2000–5000 ms), the filling of the ITI (Experiment 3 used an unfilled ITI; all other Experiments showed a fixation cross during ITI), and the type/amount of context features (in Experiments 1–4, the representation of the number stimulus varied; in Experiment 5, the representation of the number stimulus, the color of the number stimulus and the response hand varied). All five experiments remained undecisive in the test of our main hypothesis. Because all experiments tested the same hypothesis with very similar experimental designs, we decided post hoc to pool the raw data of all experiments (total *N* = 326) and submit CSE scores to a mega-analysis (also known as *Integrative Data Analysis:* Curran & Hussong, 2009; Eisenhauer, 2021; Hussong et al., 2013) to maximize test power while keeping a more complex data structure than comparable meta-analytical approaches (Sung et al., 2014; Tierney et al., 2015). The mega-analysis tested the hypothesis identical to that tested for each individual experiment, i.e., whether the c-CSE is reduced with longer ITI delays.

### Mega-analysis

#### Analysis protocol

The mega-analysis repeated the analysis protocol of the previous experiments, with the difference that the data of all five experiments were included and the between-participants factor “experiment” was added. Please note that this additional factor was intended as a control variable and is not designed to be a valid test of differences between experiments because participants were not randomly assigned to a certain experimental condition. For reasons of brevity, we report only the main effect of the factor ‘experiment’ and its interaction with the test of the temporal decay of the c-CSE.

## Results

**Fig. 2:**
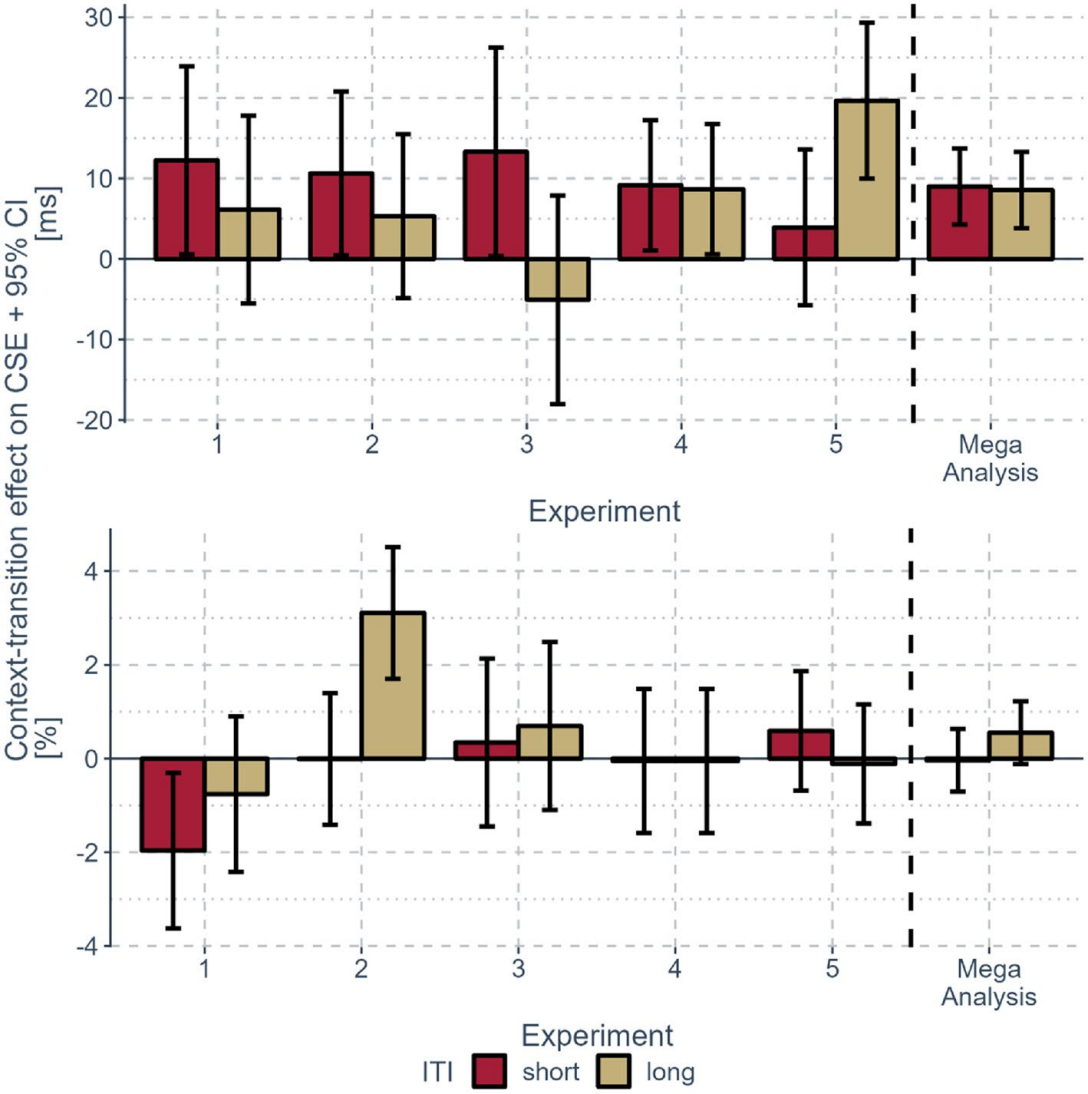
Results from Experiments 1–5 and the mega-analysis. *Note*: Context-transition effects on the CSE (c-CSEs) segmented by ITI condition (color) and experiment (*x-axis*) with the aggregated c-CSEs on the right side (separated by the dashed line). The upper panel shows the results in RTs, and the lower panel results in error rates. Error bars indicate the 95% confidence interval of paired differences (Baguley, 2012; Cousineau, 2005) (color figure online)

According to the preregistrations of the individual analyses, we excluded the first trial of each block (0.7%) and all trials following error trials (6.5%). For RT analysis, we also removed all error trials (6.5%) and trials deviating more than 3 SD from the participants’ conditional mean RT (1.3%). Mean RTs were calculated on an average of 56 observations per condition (with 16 factorial cells: four congruency transitions, two context transitions, and two ITI conditions). A visualization of the results can be found in Fig. 2, while the aggregated CSE scores can be found in Table 4.

**Table 4:** Aggregated CSEs observed in Experiments 1–5 in RTs (ms) and error rates (%) and aggregated effects of context-transition on the CSE with the lower and upper bound of the 95% within-participant confidence interval in brackets

	CSEs in	
	RTs (ms)	Error rates (%)
Short ITI duration		
Context repetition	39	1.5
Context change	30	1.5
Context-transition effect	9	0.0
Long ITI duration		
Context repetition	33	1.1
Context change	25	0.5
Context-transition effect	9	0.6

The Bayesian ANOVA for CSEs in RTs that tested whether the size of the c-CSE is reduced for longer ITIs yielded the following Bayes factors. First, Bayes factors indicated extreme evidence in favor of a main effect of the experiment factor, *BF*_10_ = 101.082 (± 0.59%). Pairwise Bayesian *t-*tests revealed decisive evidence that CSEs in Experiment 5 (*M* = 24 ms) were smaller compared to CSEs in Experiment 1 (*M* = 40 ms), *BF*_10_ = 36.142 (± 0%), and Experiment 2 (*M* = 39 ms), *BF*_10_ = 89.464 (± 0%), as well as smaller CSEs in Experiment 2 (*M* = 39 ms) compared to Experiment 4 (*M* = 29 ms), *BF*_10_ = 5.546 (± 0%). Second, there was strong evidence for a main effect of context transition, *BF*_10_ = 46.280 (± 1.71%), because CSEs were smaller in context change trials (*M* = 28 ms) than in context repetition trials (*M* = 36 ms). Third, Bayes factors remained indecisive regarding the main effect of ITI duration, *BF*_01_ = 1.183 (± 0.84%). There was strong evidence against a two-way interaction between context transition and ITI duration representing the test of our main hypothesis, *BF*_01_ = 12.330 (± 5.56%). This indicates that the c-CSE did not differ between the short and long ITI conditions. Finally, there was moderate evidence against a three-way interaction also including the experiment factor, *BF*_01_ = 6.974 (± 17.93%).

The same analysis on error rates revealed these Bayes factors. First, there was strong evidence against a main effect of experiment, *BF*_01_ = 28.184 (± 0.6%). Second, there was strong evidence against a main effect of context transition, *BF*_01_ = 12.551 (± 0.89%). Third, Bayes factors remained indecisive when testing a main effect of ITI duration, *BF*_01_ = 1.987 (± 2.73%). Furthermore, there was moderate evidence against the two-way interaction between context transition and ITI duration representing the test of our main hypothesis, *BF*_01_ = 8.634 (± 13.58%), indicating that there was no difference in the c-CSE between ITI conditions. Finally, there was strong evidence against a three-way interaction including all factors, *BF*_01_ = 24.685 (± 7.36%).

## Discussion mega-analysis

**Fig. 3:**
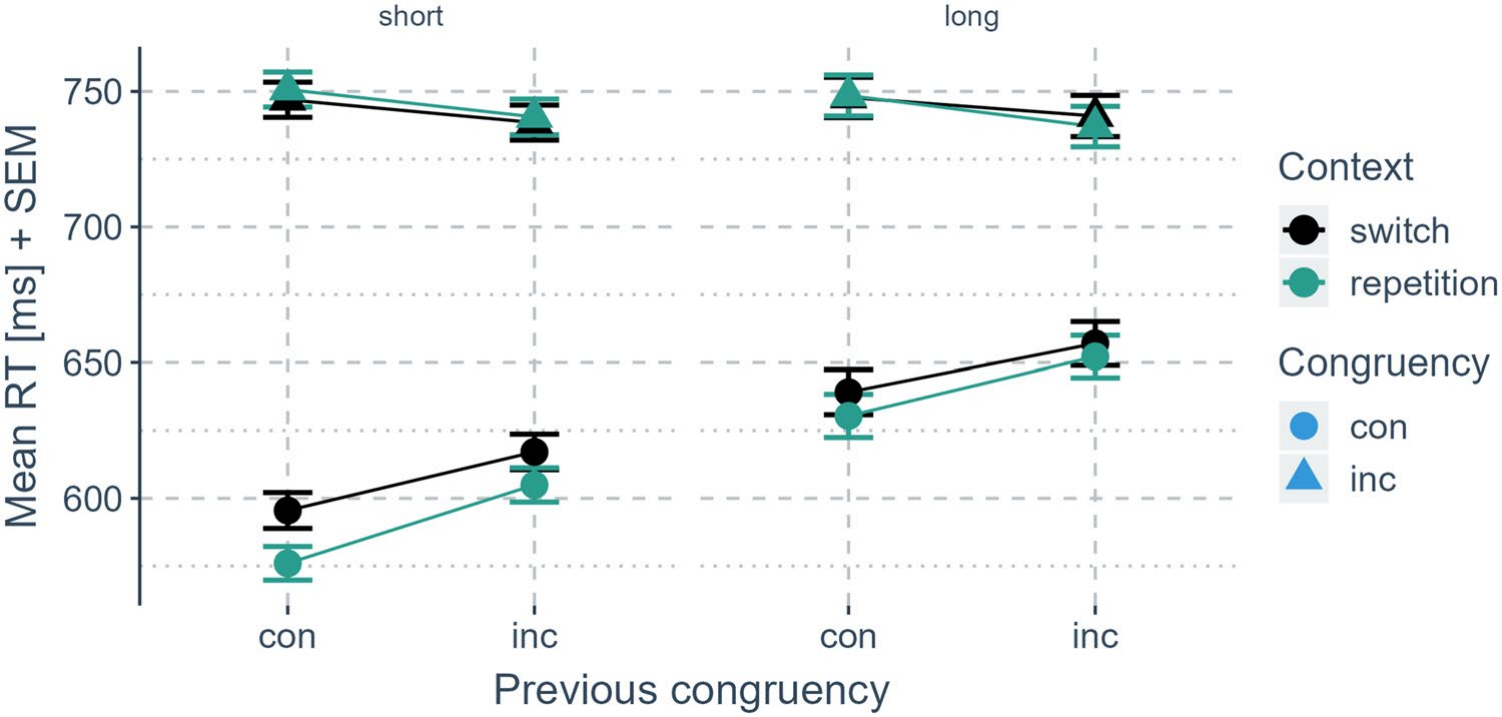
Results from the mega-analysis in mean reaction times. *Note*: Mean reaction times aggregated over all experiments segmented by congruency in the previous trial (x-axis), congruency in the current trial (shape), and context transition (color). Error bars indicate the standard error of the mean for each condition (color figure online)

To put the hypothesis to the strongest test possible here, we performed a mega-analysis analyzing a substantial sample size of 326 participants. This analysis revealed strong evidence in favor of a c-CSE replicating previous research (Dignath & Kiesel, 2021; Dignath et al., 2019; Grant et al., 2021). Most importantly, the mega-analysis provided strong evidence for the test of our main hypothesis indicating that no effect of ITI duration on the c-CSE was observed (Fig. 3).

## General discussion

The present study aimed to provide a further test of the idea that abstract control parameters are stored in memory. Going beyond previous research, we asked further whether such bindings of control states decay over time or are robust against longer retrieval delays. To probe control states, we measured CSEs in a confound-minimized design of the prime-target task and manipulated whether nominally task-irrelevant context features [in Experiments 1–4, the format of stimulus presentation (word vs. digit); in Experiment 5, the format of stimulus presentation (word vs. digit), the response hand (left vs. right) and stimulus color (blue vs. orange)] changed or were repeated across consecutive trials. We operationalized retrieval of control states as a benefit (i.e., larger CSEs) for context repetitions compared to context changes. To manipulate the length of retrieval delays, we compared the size of context-transition effects on the CSE using short and longer ITIs. The analyses of the individual experiments’ data did not provide decisive evidence when testing our main hypothesis. Furthermore, the c-CSEs observed in these experiments were surprisingly small compared to those reported in previous studies (Dignath & Kiesel, 2021; Dignath et al., 2019; Grant et al., 2021). To obtain maximal test power for the test of our main hypothesis, we decided to integrate the data of all five experiments into a single mega-analysis (*N* = 326).

This mega-analysis, which mimicked the analysis plan of the individual experiments but additionally controlled statistically for potential between-experiment differences, provided strong evidence that CSEs observed in context-repetition trials are larger than CSEs in context-change trials. Replicating previous research (Dignath et al., 2019, 2021; Grant et al., 2020), this finding suggests that context-repetitions act as a cue to retrieve abstract control states, supporting the view that internal control parameters are stored in trial-specific event files (Egner, 2014; Frings et al., 2020; Schumacher & Hazeltine, 2016). Second, the Bayesian analysis provided moderate evidence for temporally stable control states for retrieval delays of 2, 3 and 5 s.

This temporal stability is in line with a distinction between rapid memory decay for concrete stimulus–response bindings (Frings, 2011; Hommel & Frings, 2020; Pastötter et al., 2020) and a much slower memory decay for more abstract response–response bindings (Moeller & Frings, 2021). For instance, Moeller and Frings (2021) suggested that a quick decay of stimulus–response bindings might be functional because it prevents interference from previous memory episodes. In contrast, more abstract actions require the maintenance of relevant information over longer periods of time, and therefore, such higher-level bindings linking different sub actions would be more efficient if they were temporally more stable. One might speculate that a similar line of reasoning applies to control state binding. Indeed, theoretical models of cognitive control have highlighted the need to maintain abstract control settings over time to ensure adaptive goal-directed behavior (Badre, 2008). Neurophysiological data support such a hierarchical structure (see Badre & D'Esposito, 2009; also Hazy et al., 2007). Control processes based on increasingly abstract rule sets have been located along a caudal to rostral gradient in the prefrontal cortex. Intriguingly, recent data suggest that the same regions (particularly the right middle frontal gyrus) function as a central area for more durable response–response bindings (Geißler et al., 2021). Furthermore, Jiang et al. (2015) compared bindings of different abstraction levels (from concrete to abstract: stimulus–response bindings; category-response bindings; control state bindings) and found a distinct neural signature for these types of bindings whereby the allocated brain areas followed a posterior to anterior gradient with increasing abstraction level. Speculatively, bindings that encode more abstract features that control states certainly are might be more robust against temporal decay than bindings reflecting more concrete features. In sum, the present research supports an account differentiating between bindings of abstract relations and concrete features since previous studies reported a fast decay of memory for concrete stimulus–response codes (Frings, 2011; Hommel & Colzato, 2004; Hommel & Frings, 2020; Moeller & Frings, 2017; Moeller et al., 2016; Pastötter et al., 2020) that was not observed in the present data for memory for abstract control states.

Interestingly, studies in which control states preparing for task switches are paired with unique stimuli (Whitehead et al., 2020) show that such associations can be retrieved even when several minutes have elapsed after the association was formed (Whitehead et al., 2022). This is compatible with the present research suggesting that abstract control states are robust against temporal decay. In a similar design, Brosowsky and Crump (2018) showed that in a flanker task, current trial congruency can be influenced by the congruency of a trial that was presented more than 100 trials before if they are both paired with the same unique stimulus. However, they failed to find this effect in a confound-minimized experiment in which the previous and the curent trial have no overlap in the target, distractor and response. This makes it difficult to distinguish whether they observed recall of control states or stimulus–response bindings (Hommel et al., 2004). It remains to be investigated whether the binding and retrieval mechanisms studied in the present research and the more sustained associative learning of control states investigated by Whitehead et al. (2020) are independent or similar processes (e.g.Giesen et al., 2019; Moeller & Frings, 2017).

### Limitations

A limitation of the present research is the relatively smaller effect sizes of the c-CSE compared to previous findings. For instance, Dignath et al. (2019) observed c-CSEs with an absolute size of 14 ms (Exp. 1) and 24 ms (Exp. 2) and Grant et al. (2021) reported a c-CSE of 32 ms (Exp 1). In contrast, the overall c-CSE in the present research was 9 ms (in both ITI conditions). Consequently, decisive evidence for the test of our main hypothesis, that there is a temporal decay of c-CSEs but also for the to-be modulated effect (c-CSEs) was found only in the extremely high-powered, but not preregistered mega-analysis (but not in the preregistered analyses of the individual experiments). Three methodological factors could account for the smaller effect sizes of the c-CSE in the present study. First, 4 of 5 experiments in the present study were conducted online, while previous research used in-laboratory testing. Although we acknowledge that online testing might induce additional noise, studies that systematically compared in-lab and online testing have found no systematic bias and observed timing accuracy comparable to lab testing conditions (Leeuw & Motz, 2016; Pinet et al., 2017; Reimers & Stewart, 2015; Semmelmann & Weigelt, 2017). In addition, a direct comparison between Exp. 1 that was conducted in the lab and Exp. 2–5 that were conducted online provided no indication for a difference between in-lab and online studies. Second, the effect sizes of previous research might represent an overly optimistic estimate of the ‘true’ effect size. Indeed, research on the so-called ‘decline effect’ suggests that effect sizes tend to decrease with increasing years from the first publication of an effect, although the reasons for this decline effect have been debated (see e.g. Lilienfeld & Waldman, 2017). The third factor, which appears most relevant to us, could be due to overall longer delays between trials. Although ITI duration does not seem to have a specific effect on the c-CSE, it could be that overall, longer pauses during trials facilitate mind-wandering, task disengagement and possibly multitasking. Consequently, mind wandering and related off-task behavior during longer waiting periods might have interfered with the encoding and retrieval of control states. For instance, Whitehead et al. (2021) reported impaired encoding of control states in task switching during episodes of mind wandering. Relatedly, Moeller and Frings (2014) found that inattention to retrieval cues impaired retrieval of bindings. Future research could assess these speculations more systematically, for instance, by adding tests of attentiveness to binding and retrieval trials in a comparably strenuous experimental setup.

### Conclusion

A mega-analysis integrating the data of five experiments (which provided inconclusive evidence when analyzed individually) found that the c-CSE is robust against temporal delays of multiple seconds. This extends recent accounts such as the BRAC framework, which is concerned with transient memory across subsequent trials (Frings et al., 2015), pointing toward a possible link with associative theories of control that describe a more sustained learning of control (e.g., Abrahamse et al., 2016). However, since the observed c-CSEs were relatively small in the present research, future studies could use alternative paradigms (e.g., Grant et al., 2020) to provide a more detailed picture of how control state bindings play out over time.

### Electronic supplementary material

Below is the link to the electronic supplementary material.

Supplementary file 1 (DOCX 41 KB)

## Data Availability

Data, code, and materials are available on OSF (https://osf.io/k8752/).

## Appendix

Before our main analysis, we wanted to validate whether the paradigm produced CSEs. For this purpose, we analyzed only context repetitions with short ITIs, a condition that mirrors previous research that has found robust CSEs. A Bayesian ANOVA was computed with the within factors current congruency [congruent vs. incongruent] and previous congruency [congruent vs. incongruent] and participant as random factor using mean RTs as dependent variables. In the mega-analysis, we additionally included the between-participant factor “experiment”.

### Experiment 1

The Bayesian ANOVA testing for meaningful CSEs in RTs in the context transition condition with short ITIs revealed the following Bayes factors for main effects. First, there was extreme evidence for a main effect of current congruency, *BF*_10_ = 5.540 × 10^47^ (± 1.31%), because RTs were faster in congruent trials (*M* = 563 ms) than in incongruent trials (*M* = 723 ms). Second, there was moderate evidence against a main effect of previous congruency, *BF*_01_ = 3.376 (± 1.84%). A CSE was represented by extreme evidence for the two-way interaction of current and previous congruency, *BF*_10_ = 388.823 (± 52.47%), because congruency effects were smaller after incongruent (*M* = 136 ms) than after congruent trials (*M* = 184 ms).

The same analysis on error rates revealed the following Bayes factors. First, there was extreme evidence for a main effect of current congruency, *BF*_10_ = 129,153.6 (± 0.79%), because error rates were lower in congruent trials (*M* = 5.6%) than in incongruent trials (*M* = 9.4%). Second, there Bayes factors remained indecisive regarding the main effect of previous congruency. Additionally, Bayes factors for the two-way interaction between current and previous congruency remained indecisive.

### Experiment 2

Bayesian ANOVA testing for meaningful CSEs in RTs in the context transition condition with short ITIs revealed these Bayes factors for main effects. First, there was extreme evidence for a main effect of current congruency, *BF*_10_ = 2.854 × 10^70^ (± 1.31%), because RTs were faster in congruent (*M* = 555 ms) than in incongruent trials (*M* = 720 ms). Second, there was moderate evidence against a main effect of previous congruency, *BF*_01_ = 5.900 (± 1.74%). A CSE was represented by extreme evidence for the two-way interaction of current and previous congruency, *BF*_10_ = 83,096.68 (± 53.36%), because congruency effects were smaller after incongruent (*M* = 139 ms) than after congruent trials (*M* = 190 ms).

The same analysis on error rates revealed the following Bayes factors. First, there was extreme evidence for a main effect of current congruency, *BF*_10_ = 466,548.6 (± 0.8%), because error rates were lower in congruent (*M* = 4.4%) than in incongruent trials (*M* = 7.1%). Second, there was moderate evidence against a main effect of previous congruency, *BF*_01_ = 3.492 (± 1.02%). Additionally, Bayes factors for the two-way interaction between current and previous congruency remained indecisive.

### Experiment 3

Bayesian ANOVA testing for meaningful CSEs in RTs in the context transition condition with short ITIs revealed these Bayes factors for main effects. First, there was extreme evidence for a main effect of current congruency, *BF*_10_ = 1.261 × 10^72^ (± 1.32%), because RTs were faster in congruent (*M* = 587 ms) than in incongruent trials (*M* = 754 ms). Second, there was moderate evidence against a main effect of previous congruency, *BF*_01_ = 6.363 (± 1.81%). A CSE was represented by extreme evidence for the two-way interaction of current and previous congruency, *BF*_10_ = 5013.562 (± 53.47%), because congruency effects were smaller after incongruent (*M* = 144 ms) than after congruent trials (*M* = 190 ms).

The same analysis on error rates revealed the following Bayes factors. First, there was extreme evidence for a main effect of current congruency, *BF*_10_ = 10,018.18 (± 0.8%), because error rates were lower in congruent (*M* = 4.7%) than in incongruent trials (*M* = 6.9). Second, there was moderate evidence for a main effect of previous congruency, *BF*_10_ = 5.603 (± 1%), because error rates were lower in trials following congruent trials (*M* = 5.1%) than in trials following incongruent trials (*M* = 6.5%). Additionally, Bayes factors for the two-way interaction between current and previous congruency remained indecisive.

### Experiment 4

Bayesian ANOVA testing for meaningful CSEs in RTs in the context transition condition with short ITIs revealed these Bayes factors for main effects. First, there was extreme evidence for a main effect of current congruency, *BF*_10_ = 1.553 × 10^76^ (± 1.32%), because RTs were faster in congruent (*M* = 540 ms) than in incongruent trials (*M* = 670 ms). Second, there was moderate evidence against a main effect of previous congruency, *BF*_01_ = 6.111 (± 1.79%). A CSE was represented by extreme evidence for the two-way interaction of current and previous congruency, *BF*_10_ = 267.712 (± 53.35%), because congruency effects were smaller after incongruent (*M* = 135 ms) than after congruent trials (*M* = 168 ms).

The same analysis on error rates revealed the following Bayes factors. First, there was extreme evidence for a main effect of current congruency, BF10 = 1.299 × 10^10^ (± 0.8%), because error rates were lower in congruent (M = 4.2%) than in incongruent trials (M = 8.0%). Second, Bayes factors remained indecisive in regard to a main effect of previous congruency. Finally, there was extreme evidence for the two-way interaction between current and previous congruency, *BF*_10_ = 5.614 × 10^9^ (± 9.83%), because congruency effects were smaller after incongruent (*M* = 3.1%) than after congruent trials (*M* = 4.5%).

### Experiment 5

Bayesian ANOVA testing for meaningful CSEs in RTs in the context transition condition with short ITIs revealed the following Bayes factors for main effects. First, there was extreme evidence for a main effect of current congruency, *BF*_10_ = 1.604 × 10^109^ (± 1.32%), because RTs were faster in congruent (*M* = 644 ms) than in incongruent trials (*M* = 786 ms). Second, there was moderate evidence against a main effect of previous congruency, *BF*_01_ = 5.693 (± 1.96%). A CSE was represented by extreme evidence for the two-way interaction of current and previous congruency, *BF*_10_ = 154.036 (± 53.44%), because congruency effects were smaller after incongruent (*M* = 129 ms) than after congruent trials (*M* = 156 ms).

The same analysis on error rates revealed the following Bayes factors. First, there was extreme evidence for a main effect of current congruency, BF10 = 3.740 × 10^16^ (± 0.81%), because error rates were lower in congruent (M = 5.6%) than in incongruent trials (M = 10.0%). Second, Bayes factors remained indecisive in regard to a main effect of previous congruency. Finally, Bayes factors for the two-way interaction between current and previous congruency remained indecisive.

### Mega-analysis

The Bayesian ANOVA testing for CSEs in RTs in the context repetition condition with short ITIs revealed the following Bayes factors. First, the Bayes factor indicated extreme evidence for a main effect of experiment, *BF*_10_ = 49,651.74 (± 0.68%). Pairwise Bayesian *t-*tests revealed decisive evidence for faster mean RTs in Experiment 4 (*M* = 614 ms) compared to Experiment 2 (*M* = 658 ms), *BF*_10_ = 10.411 (± 0%), and compared to Experiment 3 (*M* = 669 ms), *BF*_10_ = 35.379 (± 0%). Additionally, we found decisive evidence for slower mean RTs in Experiment 5 (*M* = 712 ms) compared to Experiment 1 (*M* = 641 ms), *BF*_10_ = 13.325 (± 0%), compared to Experiment 2 (*M* = 658 ms), *BF*_10_ = 15.156 (± 0%), and compared to Experiment 4 (*M* = 614 ms), *BF*_10_ = 16,249.23 (± 0%). Second, there was extreme evidence for a main effect of current congruency, *BF*_10_ = 8.966 × 10^380^ (± 2.02%), because RTs were faster in congruent (*M* = 589 ms) than in incongruent trials (*M* = 746 ms). Third, there was moderate evidence against a main effect of previous congruency, *BF*_01_ = 3.747 (± 0.84%). Most importantly, there was extreme evidence for the two-way interaction between current and previous congruency reflecting the CSE, *BF*_10_ = 5.992 × 10^16^ (± 4.71%), indicating that congruency effects were smaller after incongruent trials (*M* = 137 ms) than after congruent trials (*M* = 166 ms). There was strong evidence against a three-way interaction also including the experiment factor, *BF*_01_ = 11.922 (± 12.96%), indicating that the CSE effect did not differ between experiments.

The same analysis in error rates revealed these Bayes factors. First, Bayes factors for an effect of the factor experiment remained indecisive. Second, there was extreme evidence for a main effect of current congruency, *BF*_10_ = 1.899 × 10^45^ (± 0.84%), because error rates were lower in congruent trials (*M* = 5.0%) than in incongruent trials (*M* = 8.5%). Third, there was extreme evidence for a main effect of previous congruency, *BF*_10_ = 894.473 (± 2.71%), because error rates were lower in trials following incongruent trials (*M* = 6.2%) than in trials following congruent trials (*M* = 7.3%). There was strong evidence for the two-way interaction between current and previous congruency representing the CSE, *BF*_10_ = 14.785 (± 13.47%), indicating that congruency effects were smaller after incongruent (*M* = 2.7%) than after congruent trials (*M* = 4.1%). Finally, Bayes factors showed extreme evidence against a three-way interaction including the experiment factor *BF*_01_ = 107.946 (± 7.47%), indicating that the error CSE did not deviate from experiment to experiment.

## Footnotes

1 For Experiment 2–5, we increased the sample size in batches of 30 participants and tested our main hypothesis under a Bayesian framework. If a decisive Bayes factor (smaller than 1/6 or larger than 6) was observed, we would stop data collection, elsewise we would continue. In Experiment 5, we would start with a minimum sample size of 100 participants to avoid accumulation of misleading evidence in smaller minimum sample sizes as suggested by Schönbrodt et al. (2016). Please note, that while we report in the manuscript the Bayes factors resulting from Bayesian ANOVAs, the stopping rule was applied based on the Bayes factor resulting from Bayesian *t*-test which was the originally preregistered approach (see “Open Science and Transparency”-statement). The Bayesian ANOVA model uses a different approach to calculate prior distributions than the Bayesian *t*-test (see Rouder et al., 2012; Rouder et al., 2009). Therefore, Bayes factors resulting from the Bayesian ANOVA differ (i.e., turned out to be more conservative) from the Bayes factors that were calculated with Bayesian *t*-tests as the criterion for the stopping rule.

## Declarations

### Open science and transparency

The registrations for all experiments can be found on OSF (https://osf.io/k8752/registrations). We deviated from the preregistered analysis plans for two reasons. First, for Experiments 1 and 2, we preregistered a frequentist approach (repeated-measures ANOVA) but decided to switch to a Bayesian framework. This allowed us to compare evidence for a model including an effect of temporal decay on the c-CSE against a null model, and we avoided violations of *null hypothesis significance testing* assumptions by repeated testing in the mega-analysis. For experiments 3 and 4, we preregistered to test our main hypothesis with a Bayesian dependent measures *t*-test contrasting difference scores (c-CSE in long ITIs vs. c-CSE in short ITIs). Upon a reviewer’s suggestion, we decided to switch from a Bayesian *t*-test to a Bayesian ANOVA that includes by-participant random intercepts since this more closely followed the preregistered analysis plans for experiments 1 and 2 and allowed us to test for other effects of interest, such as CEs, CSEs, and c-CSEs, in a single analysis. This analysis protocol was preregistered for experiment 5 and applied for all other individual analyses as well as for the mega-analysis. The applied model adhered closely to the originally preregistered analysis plan using the same factors and dependent variables as well as including by-participant random effects. Bayes factors resulting from the Bayesian ANOVA turned out to be more conservative than Bayes factors from the Bayesian *t*-tests, which were used for the stopping rule in data collection (see footnote on p. 9). The results of the preregistered analysis plan can be found in the online supplement.
