# Supplementary material for: Memory for abstract control states does not decay with increasing retrieval delays
Source: Psychol Res. 2023 Aug 24;88(2):547–61. doi: 10.1007/s00426-023-01870-4 (PMC10858070; doi:10.1007/s00426-023-01870-4)
Supplement: Supplementary file 1 — (DOCX 41 KB) [file 426_2023_1870_MOESM1_ESM.docx]

## Online Supplement

### Results of individual Bayesian analyses, separately for each Experiment

### Experiment 1

According to our preregistration, we excluded the first trial of each block (0.8 %) and all trials following error trials (7.1 %). For RT analysis, we also removed all error trials (7.2 %) and trials deviating more than 3 SD from this subjects conditional mean RT (1.3 %). Mean RTs were calculated on an average of 55 observations (*SD* = 5.5) per condition (with 16 factorial cells: four congruency transitions, two context transitions, and two ITI conditions).

**CSE validation.** The ANOVA testing for meaningful CSEs in RTs in the context repetition condition with short ITIs revealed two main effects. First, there was a main effect of current congruency, F(1, 44) = 3841.58, p < .001, ηp2= .872, because RTs were faster in congruent (M = 563 ms) than in incongruent trials (M = 723 ms). Second, there was a main effect of previous congruency, F(1, 44) = 19.97, p < .001, ηp2= .312, because RTs were faster in trials following congruent trials (M = 635 ms) than in trials following incongruent trials (M = 652 ms). A significant CSE was represented by the two-way interaction of current and previous congruency, F(1, 44) = 79.96, p < .001, ηp2= .645, because congruency effects were smaller after incongruent (M = 136 ms) than after congruent trials (M = 184 ms).

The same analysis on error rates revealed two main effects. First, there was a main effect of current congruency, F(1, 44) = 21.45, p < .001, ηp2= .328, because error rates were lower in congruent (M = 5.6 %) than in incongruent trials (M = 9.4). Second, there was a main effect of previous congruency, F(1, 44) = 9.43, p = .004, ηp2= .176, because error rates were lower in trials following incongruent trials (M = 6.6 %) than in trials following incongruent trials (M = 8.4 %). No other effect was significant (p >= .293).

**Time course of the c-CSE.** The ANOVA testing our main hypothesis, the temporal decay of CSE context modulations for RTs, revealed no significant effects (p >= .099). The two-way interaction between context transition and ITI duration representing the test of our main hypothesis was not significant, F(1, 44) < 1, p = .600. Bayes Factors for the test of the main hypothesis indicated moderate evidence in favor of the null model, i.e. no difference in the context modulation of the CSE between the short and long ITI condition (BF01 = 5.424).

The same analysis on error rates revealed no significant effects as well (p >= .107). The two-way interaction between context transition and ITI duration representing the test of our main hypothesis was not significant, F(1, 44) < 1, p = .467. Bayes Factors for the test of the main hypothesis indicated moderate evidence in favor of the null model, i.e. no difference in the context modulation of the CSE between the short and long ITI condition (BF01 = 4.805).

### Experiment 2

The analysis plan for Experiment 2 was identical to Experiment 1. According to the preregistration, we excluded the first trial of each block (0.8 %) and all trials following error trials (5.6 %). For RT analysis we also removed all error trials (5.6 %) and trials deviating more than 3 SD from this subjects conditional mean RT (1.4 %). Mean RTs were calculated on an average of 56 observations (*SD* = 5.0) per condition.

**CSE validation.** The ANOVA testing for meaningful CSEs in RTs in the context repetition condition with short ITIs revealed two main effects. First, there was a main effect of current congruency, F(1, 59) = 518.11, p < .001, ηp2= .898, because RTs were faster in congruent (M = 555 ms) than in incongruent trials (M = 720 ms). Second, there was a main effect of previous congruency, F(1, 59) = 7.68, p = .007, ηp2= .115, because RTs were faster in trials following congruent trials (M = 655 ms) than in trials following incongruent trials (M = 642 ms). A significant CSE was represented by the two-way interaction of current and previous congruency, F(1, 59) = 71.88, p < .001, ηp2= .549, because congruency effects were smaller after incongruent (M = 139 ms) than after congruent trials (M = 190 ms).

The same analysis on error rates revealed a main effect of current congruency, F(1, 59) = 24.04, p < .001, ηp2= .290, because error rates were lower in congruent (M = 4.4 %) than in incongruent trials (M = 7.1). No other effect was significant (p >= .155).

**Time course of the c-CSE.** The ANOVA testing our main hypothesis, the temporal decay of CSE context modulations for RTs, revealed a significant main effect of ITI duration, F(1, 59) = 4.87, p = .031, ηp2= .076, because CSEs were smaller in the long ITI condition (M = 31.3 ms) than in the short ITI condition (M = 46.6 ms). The two-way interaction between context transition and ITI duration representing the test of our main hypothesis was not significant, F(1, 59) < 1, p = .604. Bayes Factors for the test of the main hypothesis indicated moderate evidence in favor of the null model, i.e. no difference in the context modulation of the CSE between the short and long ITI condition (BF01 = 6.219). The remaining effect of context transition was not significant (p = .151).

The same analysis on error rates revealed a significant main effect of context transition, F(1, 59) = 4.60, p = .036, ηp2= .072, since CSEs were larger in context repetition trials (M = 1.4 %) than in context change trials (M = -0.1 %). Further, we observed a two-way interaction between context transition and ITI duration, which tests our main hypothesis, F(1, 59) = 4.90, p = .031, ηp2= .077, indicating that the modulation of the CSE by context transitions was larger in blocks with long ITIs (Δ = 3.1 %) than in blocks with short ITIs (Δ = 0 %). Please note that this effect goes in the opposite direction as expected. The corresponding Bayes Factors remained undecisive. The remaining effect of ITI duration was not significant (p >= .151).

### Experiment 3

The analysis plan for Experiment 3 was identical to the previous experiments. According to the preregistration, we excluded the first trial of each block (0.8 %) and all trials following error trials (5.9 %). For RT analysis we also removed all error trials (5.9 %) and trials deviating more than 3 SD from this subjects conditional mean RT (1.4 %). Mean RTs were calculated on an average of 56 observations (*SD* = 4.9) per condition.

**CSE validation.** The ANOVA testing for meaningful CSEs in RTs in the context repetition condition with short ITIs revealed two main effects. First, there was a main effect of current congruency, F(1, 59) = 503.59, p < .001, ηp2= .895, because RTs were faster in congruent (M = 587 ms) than in incongruent trials (M = 754 ms). Second, there was a main effect of previous congruency, F(1, 59) = 6.09, p = .017, ηp2= .094, because RTs were faster in trials following congruent trials (M = 667 ms) than in trials following incongruent trials (M = 674 ms). A significant CSE was represented by the two-way interaction of current and previous congruency, F(1, 59) = 56.59, p < .001, ηp2= .490, because congruency effects were smaller after incongruent (M = 144 ms) than after congruent trials (M = 190 ms).

The same analysis on error rates revealed two main effects. First, there was a main effect of current congruency, F(1, 59) = 17.86, p < .001, ηp2= .232, because error rates were lower in congruent (M = 4.7 %) than in incongruent trials (M = 6.9). Second, there was a main effect of previous congruency, F(1, 59) = 13.35, p < .001, ηp2= .184, because error rates were lower in trials following incongruent trials (M = 5.1 %) than in trials following incongruent trials (M = 6.5 %). A significant CSE was represented by the two-way interaction of current and previous congruency, F(1, 59) = 7.03, p = .010, ηp2= .106, because congruency effects were smaller after incongruent (M = 1.2 %) than after congruent trials (M = 3.2 %).

**Time course of the c-CSE.** The ANOVA testing our main hypothesis, the temporal decay of CSE context modulations for RTs, revealed no significant effects (p >= .161). The two-way interaction between context transition and ITI duration representing the test of our main hypothesis was not significant, F(1, 59) < 1, p = .161. Bayes Factors for the test of the main hypothesis indicated anecdotal evidence in favor of the null model, i.e. no difference in the context modulation of the CSE between the short and long ITI condition (BF01 = 2.741).

The same analysis on error rates revealed a significant main effect of ITI duration, F(1, 59) = 8.34, p = .005, ηp2= .124, since CSEs were larger in blocks with smaller ITIs (M = 0.0 %) than in blocks with short it is (M = 1.9 %). The two-way interaction between context transition and ITI duration representing the test of our main hypothesis was not significant, F(1, 59) < 1, p = .845. Bayes Factors for the test of the main hypothesis indicated moderate evidence in favor of the null model, i.e. no difference in the context modulation of the CSE between the short and long ITI condition (BF01 = 6.951). The remaining effect of context transition was not significant (p = .428).

### Experiment 4

The analysis plan for Experiment 4 was identical to the previous experiments. According to the preregistration, we excluded the first trial of each block (0.8 %) and all trials following error trials (5.8 %). For RT analysis we also removed all error trials (5.7 %) and trials deviating more than 3 SD from this subjects conditional mean RT (1.4 %). Mean RTs were calculated on an average of 56 observations (*SD* = 5.9) per condition.

**CSE validation.** The ANOVA testing for meaningful CSEs in RTs in the context repetition condition with short ITIs revealed revealed two main effects. First, there was a main effect of current congruency, F(1, 60) = 515.18, p < .001, ηp2= .896, because RTs were faster in congruent (M = 540 ms) than in incongruent trials (M = 670 ms). Second, there was a main effect of previous congruency, F(1, 60) = 8.44, p = .005, ηp2= .123, because RTs were faster in trials following congruent trials (M = 613 ms) than in trials following incongruent trials (M = 620 ms). A significant CSE was represented by the two-way interaction of current and previous congruency, F(1, 59) = 56.73, p < .001, ηp2= .486, because congruency effects were smaller after incongruent (M = 135 ms) than after congruent trials (M = 168 ms).

The same analysis on error rates revealed two main effects. First, there was a main effect of current congruency, F(1, 59) = 34.65, p < .001, ηp2= .366, because error rates were lower in congruent (M = 4.2 %) than in incongruent trials (M =8.0). Second, there was a main effect of previous congruency, F(1, 59) = 7.25, p = .009, ηp2= .176, because error rates were lower in trials following incongruent trials (M = 5.6 %) than in trials following incongruent trials (M = 6.5 %). No other effect was significant (p >= .081).

**Time course of the c-CSE.** The ANOVA testing our main hypothesis, the temporal decay of CSE context modulations for RTs, revealed a significant main effect of context transition, F(1, 60) = 4.20, p = .045, η_p_2= .065, since CSEs were larger in context repetition trials (*M* = 32.3 ms) than in context change trials (*M* = 23.4 ms). The two-way interaction between context transition and ITI duration representing the test of our main hypothesis was not significant, F(1, 60) < 1, p = .954. Bayes Factors for the test of the main hypothesis indicated moderate evidence in favor of the null model, i.e. no difference in the context modulation of the CSE between the short and long ITI condition (*BF_01_* = 7.124). The remaining effect of ITI duration was not significant (*p* = .828).

The same analysis on error rates revealed no significant effects (*p* >= .225). The two-way interaction between context transition and ITI duration representing the test of our main hypothesis was not significant, F(1, 60) < 1, p > .999. Bayes Factors for the test of the main hypothesis indicated moderate evidence in favor of the null model, i.e. no difference in the context modulation of the CSE between the short and long ITI condition (*BF_01_* = 7.136).

### Experiment 5

The analysis plan for Experiment 5 was identical to the previous experiments. According to the preregistration, we excluded the first trial of each block (0.4 %) and all trials following error trials (7.8 %). For RT analysis we also removed all error trials (7.8 %) and trials deviating more than 3 SD from this subjects conditional mean RT (1.1 %). Mean RTs were calculated on an average of 55 observations (*SD* = 7.8) per condition.

**CSE validation.** The ANOVA testing for meaningful CSEs in RTs in the context transition condition with short ITIs revealed the following main effects. First, there was a main effect of current congruency, F(1, 100) = 663.70, p < .001, ηp2= .869, because RTs were faster in congruent (M = 644 ms) than in incongruent trials (M = 786 ms). Second, there was a main effect of previous congruency, F(1, 100) = 15.21, p < .001, ηp2= .132, because RTs were faster in trials following congruent trials (M = 709 ms) than in trials following incongruent trials (M = 718 ms). A significant CSE was represented by the two-way interaction of current and previous congruency, F(1, 100) = 34.39, p < .001, ηp2= .256, because congruency effects were smaller after incongruent (M = 129 ms) than after congruent trials (M = 156 ms).

The same analysis on error rates revealed two main effects. First, there was a main effect of current congruency, F(1, 100) = 53.65, p < .001, ηp2= .349, because error rates were lower in congruent (M = 5.6 %) than in incongruent trials (M = 10.0 %). Second, there was a main effect of previous congruency, F(1, 100) = 8.44, p = .005, ηp2= .078, because error rates were lower in trials following incongruent trials (M = 8.2 %) than in trials following incongruent trials (M = 9.4 %). No other effect was significant (p >= .081). Finally, there was a significant two-way interaction of current and previous congruency, F(1,100) = 4.16 p = .044, ηp2= .040.

**Time course of the c-CSE.** The ANOVA testing our main hypothesis, the temporal decay of CSE context modulations for RTs, revealed a significant main effect of context transition, F(1, 100) = 6.28, p = .014, η_p_2= .060, since CSEs were larger in context repetition trials (*M* = 36.3 ms) than in context change trials (*M* = 24.1 ms). The two-way interaction between context transition and ITI duration representing the test of our main hypothesis was not significant, F(1, 100) < 1, p = .505. Bayes Factors for an undirected test of the main hypothesis remained undecisive, but indicated moderate evidence in favor of the null model, i.e. no difference in the context modulation of the CSE between the short and long ITI condition (*BF_01_* = 22.551), when using a directed which seems reasonable since surprisingly the c-CSE became descriptively larger in the long ITI condition. The remaining effect of ITI duration was not significant (*p* = .422).

The same analysis on error rates revealed no significant effects (*p* >= .422). The two-way interaction between context transition and ITI duration representing the test of our main hypothesis was not significant, F(1, 100) < 1, p > .505. Bayes Factors for the test of the main hypothesis indicated moderate evidence in favor of the null model, i.e. no difference in the context modulation of the CSE between the short and long ITI condition (*BF_01_* = 2.584).
